# *BCL2* genotypes and prostate cancer survival

**DOI:** 10.1007/s00066-017-1126-9

**Published:** 2017-04-10

**Authors:** Wilfried Renner, Uwe Langsenlehner, Sabine Krenn-Pilko, Petra Eder, Tanja Langsenlehner

**Affiliations:** 1 Clinical Institute of Medical and Chemical Laboratory Diagnostics, Medical University of Graz, 8036 Graz, Austria; 2 Division of Internal Medicine, GKK Outpatient Department, Graz, Austria; 3 Department of Therapeutic Radiology and Oncology, Medical University of Graz, Graz, Austria; 4 Department of Internal Medicine I, University Hospital Würzburg, Würzburg, Germany

**Keywords:** Apoptosis, Genetics, Polymorphism, Oncogene, Radiotherapy, Apoptose, Genetik, Polymorphismus, Onkogen, Strahlentherapie

## Abstract

**Purpose:**

The antiapoptotic B‑cell lymphoma 2 (*BCL2*) gene is a key player in cancer development and progression. A functional single-nucleotide polymorphism (c.-938C>A, rs2279115) in the inhibitory P2 *BCL2* gene promoter has been associated with clinical outcomes in various types of cancer. Aim of the present study was to analyze the role of *BCL2*-938C>A genotypes in prostate cancer mortality.

**Methods:**

The association between *BCL2*-938C>A (rs2279115) genotypes and prostate cancer outcome was studied within the prospective PROCAGENE study comprising 702 prostate cancer patients.

**Results:**

During a median follow-up time of 92 months, 120 (17.1%) patients died. A univariate Cox regression model showed a significant association of the CC genotype with reduced cancer-specific survival (CSS; hazard ratio, HR, 2.13, 95% confidence interval, CI, 1.10–4.12; *p* = 0.024) and overall survival (OS; HR 2.34, 95% CI 1.58–3.47; *p* < 0.001). In a multivariate Cox regression model including age at diagnosis, risk group, and androgen deprivation therapy, the CC genotype remained a significant predictor of poor CSS (HR 2.05, 95% CI 1.05–3.99; *p* = 0.034) and OS (HR 2.25, 95% CI 1.51–3.36; *p* < 0.001).

**Conclusion:**

This study provides evidence that the homozygous *BCL2*-938 CC genotype is associated with OS and C in prostate cancer patients.

## Zusammenfassung

**Zielsetzung:**

Das antiapoptotische Gen B cell lymphoma 2 (*BCL2*) spielt eine Schlüsselrolle in der Entstehung und Progression von Krebserkrankungen. Ein funktioneller Einzelnukleotid-Polymorphismus (c.-938C>A, rs2279115) im inhibitorischen P2-*BCL2*-Promotor wurde mit dem klinischen Outcome verschiedener Krebserkrankungen verknüpft. Ziel der vorliegenden Studie war die Untersuchung der Rolle von *BCL2*-938C>A-Genotypen für die Mortalität bei Patienten mit Prostatakarzinom.

**Methoden:**

Der Zusammenhang zwischen* BCL2*-938C>A-Genotypen (rs2279115) und dem Outcome bei Prostatakrebs wurde in der prospektiven PROCAGENE-Studie, die 702 Patienten mit Prostatakarzinom umfasste, untersucht.

**Ergebnisse:**

Während der medianen Nachbeobachtungszeit von 92 Monaten starben 120 (17,1 %) Patienten. In einer univariaten Cox-Regressionsanalyse zeigte sich ein signifikanter Zusammenhang zwischen dem CC-Genotyp und einer schlechteren krebsspezifischen (CCS; Hazard Ratio [HR] 2,13; 95 %-Konfidenzintervall [KI] 1,10–4,12; *p* = 0,024) und Gesamtüberlebensrate (OS; HR 2,34; 95 %-KI 1,58–3,47; *p* < 0,001). In einer multivariaten Cox-Regressionsanalyse, die das Alter bei Diagnose, die Risikogruppe sowie die Androgendeprivationstherapie beinhaltete, blieb der CC-Genotyp ein signifikanter Vorhersagemarker für ein schlechteres CCS (HR 2,05; 95 %-KI 1,05–3,99; *p* = 0,034) und OS (HR 2,25; 95 %-KI 1,51–3,36; *p* < 0,001).

**Schlussfolgerung:**

Die vorliegenden Daten zeigen, dass der homozygote *BCL2*-938-CC-Genotyp bei Patienten mit Prostatakarzinom mit dem CSS und OS assoziiert ist.

Apoptosis or programmed cell death is an evolutionarily conserved and highly organized mechanism of cell suicide for maintaining cellular homeostasis and removing senescent or potentially hazardous cells [1]. Impaired apoptosis has been related to development and progression of various cancer types [2].

B-cell lymphoma 2 (Bcl-2) family proteins are essential regulators of apoptosis and comprise both pro- and antiapoptotic members [3]. The founding member of the Bcl-2 family is encoded by the *BCL2* gene located on chromosome 18q21.3. *BCL2* expression is regulated by two distinct promoters, P1 and P2. These promoters have different functions, with P2 decreasing the activity of P1 promoter function [4].

*BCL2* itself seems to act as both an oncogene and a tumor suppressor gene in different tumor types [5]. In prostate cancer, the role of *BCL2* expression in disease progression is currently not fully understood: Stackhouse and coworkers reported a positive correlation between *BCL2* tumor staining and biochemical recurrence in prostatectomy specimens, but not in prostate biopsies [6]. Khor and coworkers observed no association between *BCL2* overexpression and prostate cancer outcome [7, 8]. Anvari and coworkers reported an association of high *BCL2* expression with higher Gleason scores (GS) and lower biochemical recurrence-free survival in patients with advanced prostate cancer undergoing androgen deprivation therapy (ADT) [9].

A functional single-nucleotide polymorphism (c.-938C>A, rs2279115) in the P2 promoter has been shown to influence *BCL2* expression. The *BCL2* -938C allele was significantly associated with increased P2 promoter activity, resulting in decreased overall *BCL2* transcriptional activity and protein expression [10, 11]. The *BCL2*-938 CC genotype has been linked to an increased risk for biochemical recurrence after radical prostatectomy [12]. In contrast to this, another study by Bachmann et al. reported an association of the *BCL2*-938 AA genotype with a worse outcome of prostate cancer patients [11].

Aim of the present study was to test a possible association between *BCL2*-938C>A genotypes and prostate cancer outcome.

## Materials and methods

The Austrian Prostate Cancer Genetics (PROCAGENE) study includes 702 prostate cancer patients recruited between January 2004 and January 2007. A detailed description has been published previously [13–15]. Briefly, PROCAGENE is a prospective study aimed at investigating genetic risk factors, functional relationships between genetic variations and clinical phenotypes, the genetically modified response to radiotherapy (radiogenomics), and the prognostic importance of genetic markers such as genetic variants in regulators of DNA repair, cell cycle, and apoptosis, including *BCL2*-938C>A genotypes [16–21].

Participants of PROCAGENE were male patients with sporadic, histologically confirmed prostate cancers treated with radiotherapy. The study population comprised 676 patients treated with curative intent. Among them, 110 patients received postoperative radiotherapy; in 27 patients (3.8%), radiation treatment was administered with palliative intent. Clinical characteristics were obtained from medical records and prostate cancer patients were stratified into low-, intermediate-, and high-risk groups according to the National Comprehensive Cancer Network (NCCN) guidelines [22].

All patients underwent three-dimensional conformal radiotherapy for prostate cancer. The clinical target volume included the entire prostate and the base of the seminal vesicles. A safety margin of 10 mm was added in all directions to create the planning target volume (PTV). High-energy photons (18 MV) were delivered in a three-field technique using an anterior and two lateral fields to encompass the PTV. A subgroup of patients (*n* = 110) received postoperative radiotherapy using high-energy photons (18 MV) in a conformal three-field technique to treat the prostate bed. All fields were treated daily, 5 days/week. The total dose prescribed to the International Commission on Radiation Units and Measurement point was 66 to 70.4 Gy delivered in 1.8–2 Gy per fraction. None of the included patients received pelvic node irradiation.

A total of 454 patients (64.7%) received neoadjuvant ADT and 153 patients (21.8%) were treated with additional adjuvant ADT. The administration of ADT was at the discretion of the treating urologists and generally recommended in intermediate- and high-risk patients. Follow-up examinations were performed at regular intervals at the Department of Therapeutic Radiology and Oncology (3-month intervals in years 1 to 3, 6‑month intervals in years 4 to 5, and 12-month intervals in years 6 to 15 after diagnosis). The administration of systemic therapy for disease recurrence was at the discretion of the treating urologist and/or medical oncologist, and included hormonal treatment and/or chemotherapy.

The study was performed according to the Austrian Gene Technology Act and was approved by the Ethical Committee of the Medical University of Graz (EK 20-248 ex 08/09). Written informed consent was obtained from all participating subjects. All subjects were Caucasian.

### Genotyping

Upon study entry, each PROCAGENE participant donated a tube of ethylenediaminetetraacetic acid (EDTA) blood, which was stored at −20 °C. Genomic DNA was prepared from whole blood using a silica membrane technology (Machery-Nagel, NucleoSpin Blood, Germany). *BCL2* genotypes were determined by fluorogenic 5′ exonuclease assays (TaqMan™; Thermo Fisher Scientific, Pittsburgh, PA, USA) with primer and probe sets designed and manufactured by Applied Biosystems (Life Tech Austria, Vienna, Austria; assay ID C___3044428_30). Endpoint fluorescence was measured in a POLARstar plate reader (BMG Labtech, Durham, NC, USA). Fluorescence data were exported into Excel format and analyzed as scatter plots. As a quality control, genotyping was repeated in 96 samples and no discrepancies were observed.

### Statistics

The study endpoints were cancer-specific survival (CSS) defined as the time from prostate cancer diagnosis to death from prostate cancer, and overall survival (OS) calculated from time of diagnoses to the date of death from any cause. Statistical analysis was done using IBM SPSS statistics 22 software (IBM Deutschland GmbH, Ehningen, Germany). Continuous variables were compared between groups by univariate analysis of variance (ANOVA). Hazard ratio (HR) and 95% confidence intervals (CI) were analyzed by Cox regression analyses. Median follow-up times were calculated according to Schemper and Smith [23]. The criterion for statistical significance was *p* < 0.05.

## Results

**Fig. 1:**
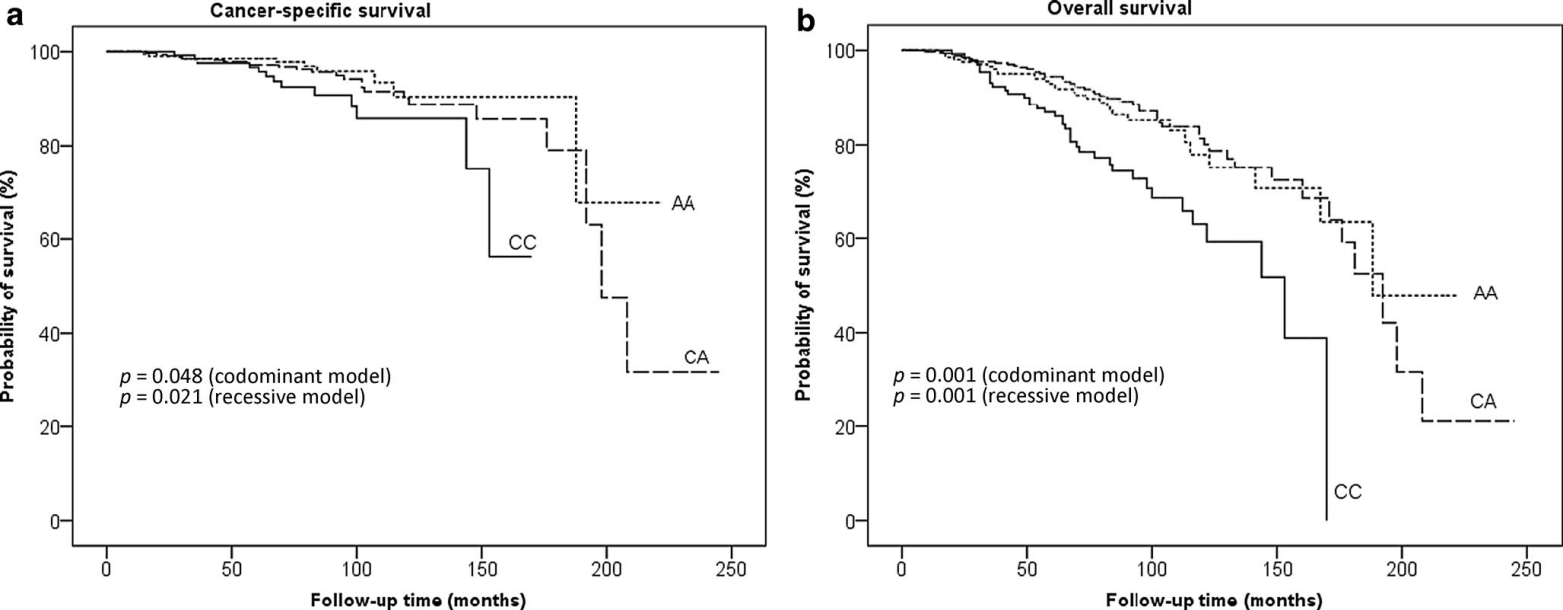
*BCL2* c.-938C>A genotypes and survival rates. **a** Cancer-specific survival: number of events and total numbers were 13/138 for the CC genotype, 24/348 for the CA genotype, and 9/215 for the AA genotype. **b** Overall survival: number of events and total numbers were 38/138 for the CC genotype, 52/348 for the CA genotype, and 31/215 for the AA genotype

*BCL2* genotypes were successfully determined in 701 patients (99.9%) of the PROCAGENE study. In the remaining subject, *BCL2* genotype was considered non-interpretable after two repeats. Genotype frequencies did not deviate from the Hardy–Weinberg equilibrium. Demographic data and genotype counts are shown in Table 1. Survival follow-up was available for all patients of the PROCAGENE study. Median follow-up time for survival was 92 months (minimum 1 month, maximum 245 months). During follow-up, 120 patients (17.1%) died, including 47 cancer-specific deaths. In a Kaplan–Meier analysis evaluated by the log-rank test, *BCL2*-938 genotypes were significantly associated with shorter CSS (*p* = 0.048; Fig. 1a) and OS (*p* < 0.001; Fig. 1b). Results from the Kaplan–Meier analysis, as well as data from a previous study in prostate cancer patients, suggested a recessive effect of the *BCL2*-938C allele on survival [9]. All further statistical tests were therefore performed comparing the CC genotype versus (CA + AA) genotypes.

**Table 1:** Demographic and genetic data of the PROCAGENE study

No. of patients (*N*)		702
Age at diagnosis (years)		68.1 ± 7.2
Stage (*n* = 655)	T1/T2 T3/T4	362 (55.3%) 293 (44.7%)
Gleason score	<7 7 >7	419 (59.7%) 134 (19.1%) 148 (21.1%)
Prostate specific antigen (PSA) level at diagnosis (ng/ml)	0–10 10–20 >20 Missing data	366 (52.1%) 164 (23.4%) 141 (20.1%) 31 (4.4%)
Risk group	Low risk Intermediate risk High risk	136 (19.4%) 181 (25.8%) 385 (54.8%)
*BCL2*-938C>A genotype	CC CA AA	137 (19.9%) 341 (49.5%) 211 (30.6%)

In Kaplan–Meier analysis, the CC genotype was significantly associated with reduced CSS (*p* = 0.021; Fig. 1a) and OS (*p* < 0.001; Fig. 1b). Furthermore, in a univariate Cox regression model, CSS (HR 2.13, 95% CI 1.10–4.12; *p* = 0.024) and OS (HR 2.34, 95% CI 1.58–3.47; *p* < 0.001) were significantly reduced for the CC genotype (Table 2). In a multivariate Cox regression model including age at diagnosis, ADT, and risk group based on PSA level, GS, and T stage, the CC genotype remained a significant predictor of poor CSS (HR 2.05, 95% CI 1.05–3.99; *p* = 0.034) and OS (HR 2.25, 95% CI 1.51–3.36; *p* < 0.001, Table 2).

**Table 2:** Univariate and multivariate Cox proportional analysis of clinical parameters for the prediction of cancer-specific and overall survival in prostate cancer patients

	Cancer-specific survival				Overall survival			
	Univariate analysis		Multivariate analysis		Univariate analysis		Multivariate analysis	
Parameter	HR (95% CI)	*p*-value*	HR (95% CI)	*p*-value*	HR (95% CI)	*p*-value*	HR (95% CI)	*p*-value*
Age at diagnosis (years)	0.99 (0.95–1.03)	0.51	1.01 (0.96–1.05)	0.75	1.02 (0.99–1.04)	0.23	1.02 (0.99–1.05)	0.21
*Risk group*								
Low risk	1	–	1	–	1	–	1	–
Intermediate risk	2.07 (0.51–8.38)	0.31	2.05 (0.50–8.37)	0.32	1.41 (0.79–2.54)	0.25	1.34 (0.74–2.42)	0.33
High risk	4.45 (1.37–14.4)	0.013	4.22 (1.28–13.9)	0.018	1.33 (0.80–2.20)	0.27	1.29 (0.76–2.16)	0.34
*Androgen deprivation therapy*								
No ADT	1	–	1	–	1	–	1	–
Neoadjuvant ADT	0.83 (0.43–1.62)	0.59	0.94 (0.47–1.86)	0.86	1.09 (0.72–1.64)	0.69	1.03 (0.67–1.57)	0.90
Neoadjuvant adjuvant ADT	0.97 (0.45–2.07)	0.93	0.80 (0.36–1.77)	0.58	1.04 (0.64–1.70)	0.86	0.92 (0.56–1.52)	0.74
*BCL2*-*938C>A genotype*								
CA + AA	1	–	1	–	1	–	1	–
CC	2.13 (1.10–4.12)	0.024	2.05 (1.05–3.99)	0.034	2.34 (1.58–3.47)	<0.001	2.25 (1.51–3.36)	<0.001

*CI c*onfidence interval, *HR* hazard ratio, *ADT* androgen deprivation therapy

*Adjustment for all factors listed in the table

Furthermore, an OS analysis in subgroups stratified by tumor stage and hormonal treatment was performed. The *BCL2* CC genotype was significantly associated with OS in the subgroup tumor stage 1–2 with hormonal treatment (HR 3.08, 95% CI 1.64–5.76; *p* < 0.001). No significant association with OS was observed in subgroups tumor stage 1–2 without hormonal treatment (HR 1.35, 95% CI 0.30–6.10; *p* = 0.70), tumor stage 3–4 with hormonal treatment (HR 2.03; 95% CI 0.89–4.65; *p* = 0.093), and tumor stage 3–4 without hormonal treatment (HR 1.23; 95% CI 0.39–3.86; *p* = 0.73).

## Discussion

In prostate cancer studies, survival is the strongest endpoint and, if available, should be preferred to other surrogate endpoints such as biochemical recurrence or development of metastases [24]. The current study found a strong association of the *BCL2*-938 CC genotype with reduced survival in prostate cancer patients. The mechanism underlying this finding is likely due to reduced *BCL2* expression in carriers of this genotype [8]. The role of *BCL2* expression in cancer development and progression is complex and still not fully understood. Depending on tumor type and disease stage, as well as therapy, *BCL2* seems to be able to act as both an oncogene and a tumor suppressor gene [5]. The overall effect of the presumably low-expression *BCL2*-938 CC genotype resulted in strongly reduced survival rates in the present cohort of prostate cancer patients.

In subgroup analyses stratified by tumor stage and hormonal treatment, the association of *BCL2* CC genotype with OS seemed to be strongest in the subgroup tumor stage –2 with hormonal treatment. Nevertheless, sample sizes of these subgroups were small and 95% CIs of HRs in different subgroups were overlapping; thus these post-hoc findings should be interpreted cautiously and require further replication. Data from the present study do not provide a plausible functional explanation for the different effect sizes of the genotype in these subgroups.

In the present study, as well as in other studies in European populations, the *BCL2*-938A allele is the common allele, whereas in Asian and Sub-Saharan African populations the C allele is more common, indicating that the C allele is the ancestral allele and the A allele is the “mutated” allele. This study observed a recessive effect of the *BCL2*-938C allele on overall survival, with reduced survival in carriers of the homozygous CC genotype [11]. Survival rates in carriers of the CA genotype were similar to those among patients with the AA genotype. The precise mechanism for this lack of a typical allele-dose effect remains to be determined.

The present results are in contrast to those of Bachmann et al., who reported reduced survival in prostate cancer patients carrying the homozygous *BCL2*-938 AA genotype [11]. The reason for this discrepancy is unclear and cannot be explained by ethnic differences. A major strength of the study by Bachmann et al. is the thorough analysis of functionality of the *BCL2* genotype, substantiating their results. Nevertheless, the small sample sizes of both the primary (*n* = 142) and the replication cohort (*n* = 148) might be regarded as a limitation of the latter study. Further studies are needed to clarify these contrasting results.

Cheaper and faster genotyping platforms allow the analysis of many gene polymorphisms in a single study. This, together with reliance on the arbitrary significance threshold of *p* < 0.05, has led to an overwhelming rate of false-positive results [25]. Correcting for multiple testing, e. g., using Bonferroni correction, reduces the rate of false-positive findings, but at the same time reduces statistical power and increases the risk of false-negative results [26, 27]. To address this problem, restriction to plausible hypotheses and potential risk factors with a high prior probability of positive association has been recommended [28]. In the current study, the authors have therefore deliberately decided to analyze only the *BCL2* gene variant with the highest prior probability for a positive association with prostate cancer mortality. The *BCL2* c.-938C>A polymorphism is the only *BCL2* variant that has been shown to influence *BCL2* expression and has been linked to prostate cancer prognosis in a previous study [10, 11].

It should be noted that the focus of the present study was germline *BCL2* genotypes, therefore all analyses were performed in non-tumor tissue. Further *BCL2* (dis-)regulation might be due other effects, such as de novo tumor mutations, which were not analyzed in the present study.

## Conclusion

This study provides evidence that the homozygous *BCL2*-938 CC genotype is strongly associated with reduced OS in prostate cancer patients.
